# First principles study of oxygen vacancy activation energy barrier in zirconia-based resistive memory

**DOI:** 10.1038/s41598-020-62270-x

**Published:** 2020-03-25

**Authors:** Ji-Hyun Hur

**Affiliations:** 1 Department of Electrical Engineering, Sejong University, 209, Neungdong-ro, Gwangjin-gu, Seoul 05006 Republic of Korea; 2 Hur Advanced Research, 96, Dongtanbanseok-ro, Hwaseong-si, Gyeonggi-do 18456 Republic of Korea

**Keywords:** Nanoscale devices, Nanoscale materials

## Abstract

Unlike experimental measurements that appeared to be quite large activation barriers, oxygen vacancies in zirconia-based resistive random access memory (ReRAM) are believed to migrate with a fairly low energy barrier, and this discrepancy has not been noticed nor seriously questioned up to date. In this paper, we work on this problem by means of first-principles calculations categorizing all the possible migration pathways by crystallographic directions. From the results, it is found that the low activation energy of oxygen vacancy that is expected from the switching characteristic of the device is originated from +2q charged oxygen vacancies in a nanometer-sized filament migrating into a particular crystallographic direction of monoclinic zirconia.

## Introduction

In recent years, oxide-based resistive random access memory (ReRAM) operating in bipolar mode has been one of the promising candidate to replace current memory devices due to its potential advantages in good scalability, high endurance of switching, and fast switching speed. Oxide-based ReRAMs usually have bi-layer oxide structures, in which a few nanometer thick, more resistive oxide layer (top layer) where actual resistance change occurs and a thicker, sub-stoichiometric base layer are sandwiched by two electrodes^1–9^. The more conductive sub-stoichiometric base layer acts as an oxygen vacancy reservoir to supply oxygen vacancies to the filament in the top layer. It is fabricated to be sub-stoichiometric to have much lower resistance than the as-deposited top layer^1–5^ that most of potential drop takes place within the top layer, allowing the switching voltage as small as possible.

Among those transition metal oxides typically deployed in oxide-based ReRAMs as resistance changing materials, zirconia (ZrO_2_) is one of the most popular one^2,4,10–13^. Besides from ReRAM, even only in semiconductor device applications, zirconia has a wide variety of applications such as high-k dielectrics in silicon or III-V metal-oxide-semiconductor field-effect transistor (MOSFET) devices^14,15^. It is known that there are mainly three phases of ZrO_2_ (a) monoclinic, at temperatures below 1700 °C, (b) tetragonal, above 1700 °C up to 2370 °C, and (c) cubic, above 2370 °C^16^. Although by addition of impurities with lower valent cations such as Y_2_O_3_ and CaO, cubic fluorite structured ZrO_2_ can be stabilized and thus various good properties of the structure like chemical inertness and high ionic conductivity can be retained even at room temperature^12^, the importance of monoclinic phase ZrO_2_ which is most stable structure in the temperature range near room temperature, could never be overlooked.

Since, in general, impurity doping is not involved in monoclinic ZrO_2_, O self-diffusion via O vacancies is almost the only way of atomic migration in the O deficient, sub-stoichiometric lattice. Several experimental measurements have been reported on O self-diffusion characteristics in monoclinic ZrO_2_ of which activation energies of O are larger than 2.4 eV^17,18^. However, from the switching model of ZrO_2_ ReRAMs^5,7^ combined with the experimental I-V curve (with the filament length of 3 nm, the lattice constant ~5.35 Å, ∼±4 V of set initiation voltage, and ±4.5 V of reset/set voltages^4,5^) we can get the activation energy of ~0.72 eV that is much lower than the experimental activation energy barriers of monoclinic ZrO_2_. In fact, the underlying principle of this discrepancy between the diffusion characteristics from pure material property and what emerged in ReRAM has never been seriously explored at all. Given the fact that resistance switching in an oxide-based ReRAM is caused by migrations of positively charged O vacancies, one thing is clear that the features regarding O migration in ReRAMs must be related with charged O vacancies. Recently, a theoretical study of O diffusivity in monoclinic ZrO_2_ by first-principle calculations was reported^19^, however, they made a conclusion that activation barrier energy is about 2 eV which clearly differs from the consensus of experimental measurements or the value expected from ZrO_2_ ReRAM operation.

In this study, we theoretically investigate the activation barrier of O vacancies in monoclinic ZrO_2_ by first-principles methods. Analyzing almost all the possibilities, we find out the pathway that determines O vacancy migration characteristics in ZrO_2_ ReRAM. Since the O activation energy measurement experiments were performed on the sub-stoichiometric specimens, we also assume O poor environment where O vacancies are the most dominant defects. From the results, we show that while the diffusivity measurements reflect migration of electrically neutral O vacancy in a bulk sample having no surface-directional preference, the activation barrier extracted from ZrO_2_ ReRAM operation comes from migration of +2q charged O vacancies through the particular crystallographic directions of monoclinic ZrO_2_ in a nanometer-sized volume called filament.

## Material model and computational details

**Figure 1:**
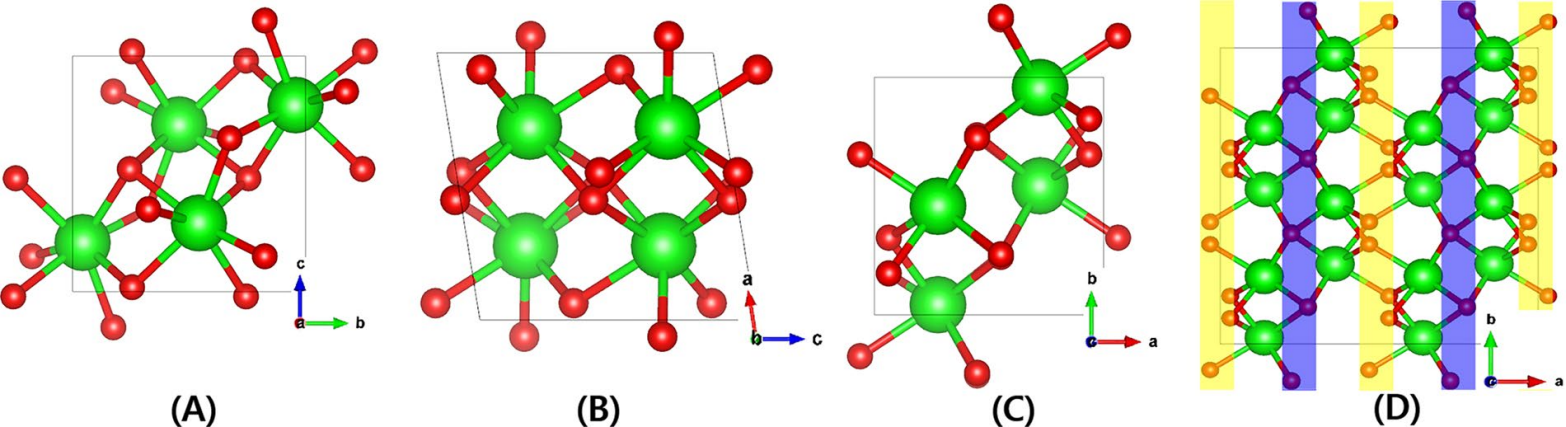
Atomic structure of the monoclinic ZrO_2_ built using VESTA v3.4.8^32^ viewed along (**A**) a-axis, (**B**) b-axis, (**C**) c-axis and (**D**) 3-fold (yellow) and 4-fold (blue) oxygens in the 2 × 2 × 2 expanded supercell viewed from c-axis. Green and red balls are Zr and O atoms respectively.

**Figure 2:**
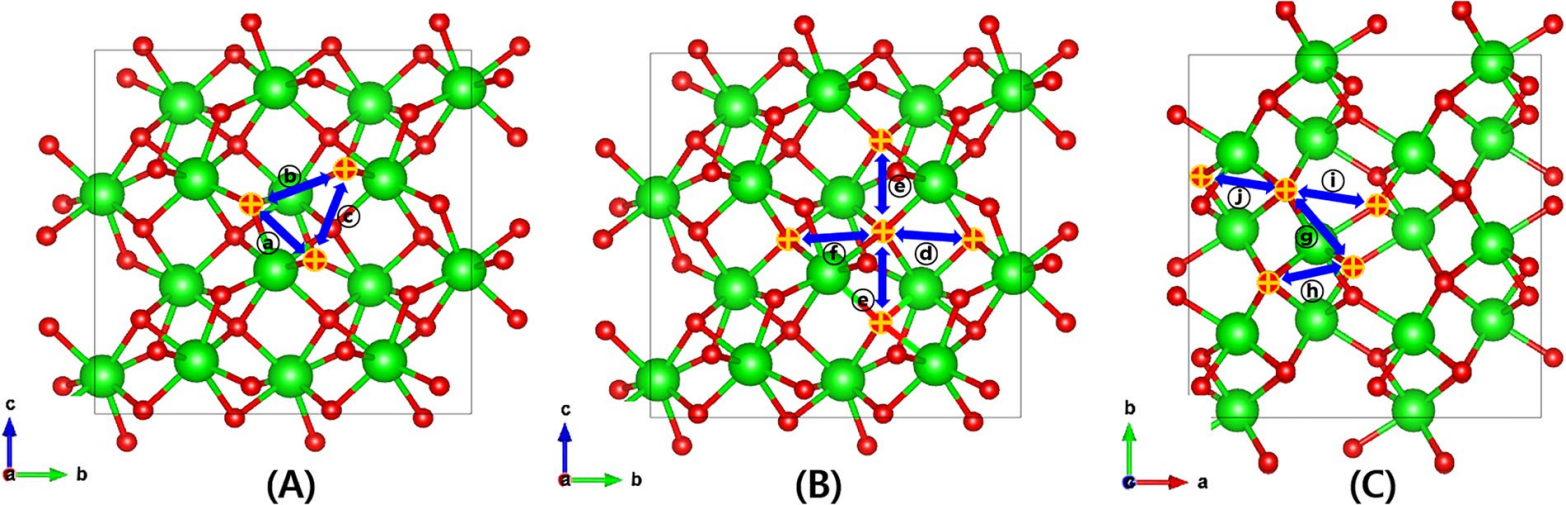
The possible O vacancy migration pathways plotted using VESTA v3.4.8^32^. (**A**) Between the 3-fold and 3-fold oxygen sites, (**B**) between the 3-fold and 4-fold oxygen sites, and (**C**) between the 4-fold and 4-fold oxygen sites. The distances between two O sites for each path measured with GGA-relaxed supercell are 2.606, 2.877, 2.809, 2.617, 2.680, 2.753, 2.992, 2.560, 3.029, and 3.287 Å for the paths ⓐ to ⓙ in order respectively.

Monoclinic ZrO_2_ has the unit cell structure of four 4-fold O and three 3-fold O atoms around one Zr atom. Figure 1 shows the unit cell of monoclinic ZrO_2_ from the three different view angles along the lattice vectors with the GGA-optimized parameters of a = 5.19 Å, b = 5.26 Å, and c = 5.35 Å which are the same with refs. ^20,21^. To clearly visualize positions of 3-fold and 4-fold O sites in the monoclinic ZrO_2_ lattice, we mark them in the 2 × 2 × 2 expanded cell (Fig. 1(D)). Since there are two types of O vacancy, we can sort out all the O vacancy migration pathways in monoclinic ZrO_2_ into three categories: migration (1) between 3-fold, (2) between 3-fold and 4-fold, and (3) between 4-fold O vacancy sites. We consider all the possible O migration pathways by analyzing 10 nearest O atoms from any O atoms and are illustratively given in Fig. 2. Every pathway is uniquely defined by the direction of migration as well as the distance between the apparent position and the O atom. We note the unique distances between O atom pairs of all the migration pathways from the GGA-relaxed cell in the figure caption. It is important that not all the possible migration paths between neighboring O sites have a practical meaning linked to the experimentally measurable diffusion properties. This is because experiments measure collective migrations of O atoms in a particular direction of a sample, thus we only count the paths continuously connected in specific directions. From now on, we confine our study on pathways that can continue in particular directions of crystal which we call ‘self-connected path’^8^. Computationally, it means O vacancy can migrate from one end of the supercell to the opposite end through the pathways. It can be figured out that for between 3-fold O migrations (Fig. 2(A)), ⓑ and ⓒ are self-connected by themselves, whereas ⓐ must be combined with either ⓑ or ⓒ to be a connected path. Likewise, for between 4-fold O migrations (Fig. 2(B)), ⓓ must be combined with ⓕ to be a connected path while ⓔ is a self-connected path by itself. Finally, for migrations between 3-fold and 4-fold O sites (Fig. 2(C)), ⓖ and ⓘ are connected with ⓗ or ⓙ to become connected pathways.

**Figure 3:**
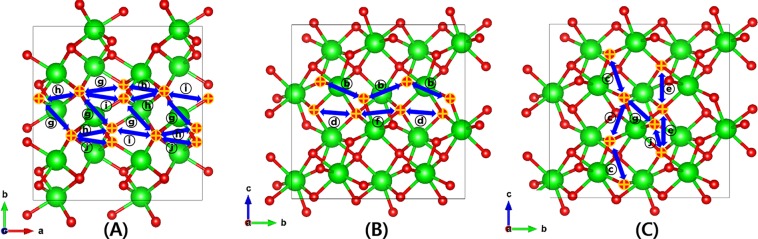
The self-connected O vacancy migration pathways built using VESTA v3.4.8^32^ classified by the migration direction to the three crystallographic axes. (**A**) (100) (ⓗ and ⓙ each combined with ⓖ or ⓘ), (**B**) (010) (ⓑ and ⓓ+ⓕ), and (**C**) (001) (ⓒ, ⓔ, ⓖ+ⓙ) directions. Some O atoms appear to be one because they overlap in viewing direction.

Here, let us consider the case of frequently occurring experimental environments: That is, interested surface of an oxide sample being exposed with a certain crystallographic direction. Because, as we have discussed so far, the specific pathways are involved with a specific O migration direction, the diffusion characteristics of the oxide are likely to change accordingly. From this point of view, it would be useful to explore how the O diffusion in monoclinic ZrO_2_ exhibits different behaviors depending on the migration direction. In Fig. 3, we present all the connected O vacancy migration pathways explained in the above sentence shown in Fig. 2 recategorized by migration directions to the three crystallographic axes of monoclinic ZrO_2_. The self-connected pathways for (100) direction are ⓗ and ⓙ each combined with ⓖ or ⓘ, ⓑ and ⓓ+ⓕ are for (010), and finally ⓒ, ⓔ, ⓖ+ⓙ constitute the self-connected pathways for (001) direction.

## O vacancy formation energy for different charge states

Since O vacancies prefer particular charge states depending on electron’s Fermi level, we need to find out formation energy of O vacancy for various charge states as a function of Fermi level. One thing to consider in calculating the defect formation energy of large bandgap materials such as ZrO_2_ is that DFT (density functional theory) calculations produce much smaller band gap than the experimental value. Setting the exchange term to hybrid functionals that mix HF (Hartree-Fock) and DFT exchange energies with some portions is widely used way to make up for this shortcoming of DFT calculations^21,22^. In the present study, we used PBE0 hybrid functional which was reported to yield the band gap of monoclinic ZrO_2_ closest to the experimental value^21^.

Because our interest is focused on relative easiness of formation of O vacancies for different charge states, we calculate the relative formation energies of O vacancies instead of absolute values that simplifies analysis by excluding the chemical potential of each atomic species. The relative formation energies of O vacancy states can be determined from the following expression^21^

1 $${{\rm{E}}}_{f}({V}_{O}^{q})={\rm{E}}({V}_{O}^{q})-{\rm{E}}({V}_{O}^{0})+q({{\rm{E}}}_{V}+{{\rm{\mu }}}_{e})+{E}_{Mad},$$

where ${\rm{E}}({V}_{O}^{q})$ is the total energy of O vacancy with charge q, ${\rm{E}}({V}_{O}^{0})$ is the total energy of neutral O vacancy, E_V_ is the valence band maximum energy, μ_e_ is the Fermi energy of electron, and E_Mad_ is the Madelung correction for the electrostatic interaction between the artificial homogeneous background charges and charged defects which is calculated to be 0.08 eV and 0.32 eV for ± q and ± 2q respectively for the current case^23^.

**Figure 4:**
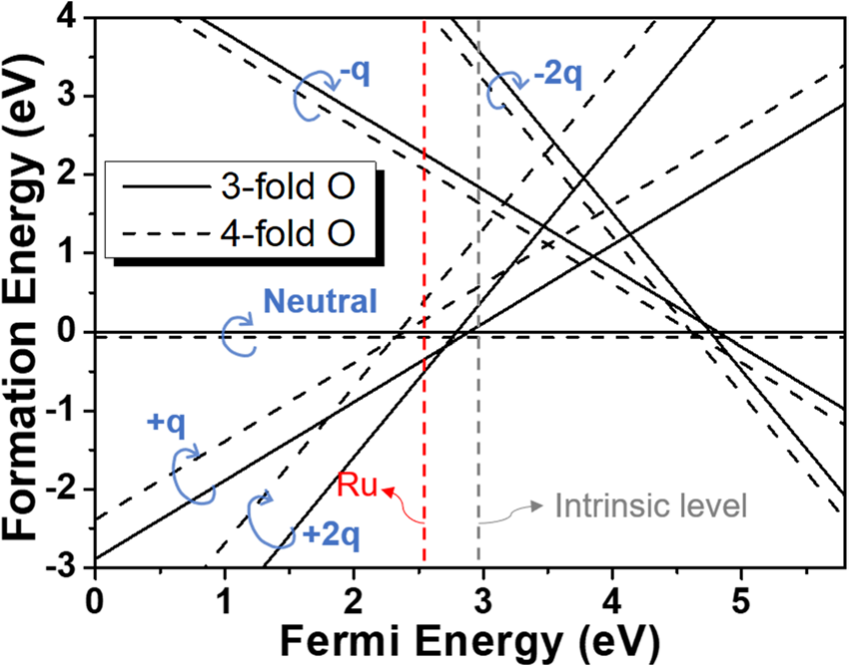
The calculated relative formation energy of the 3-fold and 4-fold O vacancies for the electrically neutral, +1, +2, −1, and −2 charged cases. The range of Fermi energy is set to be 5.8 eV which is the theoretically reported band gap of monoclinic ZrO_2_^21^. The Fermi level of Ru and intrinsic Fermi level are also shown.

We show in Fig. 4 the calculated relative formation energies of various charge states of O vacancies referenced to the that of the neutral 3-fold O vacancy. The formation energy of O vacancy with specific state is determined by the lattice energy with that kind of O vacancy and chemical potential of added (or removed) electron. For example, the formation energies of +2q and +q charged O vacancy intersect, i.e., having the same value at E_F_ ~ 3 eV, which means that the energies of the two states coincide when the chemical potential of the removed (or added) electron is about 3 eV. From the figure, it can be noticed that for Fermi energy near the intrinsic level (center of band gap) that bulk insulators normally have, O vacancies are most likely to have neutral charge state regardless of O types followed by +1 and +2 charge states that nearly coincides with the result of ref. ^24^. Under this circumstance, O vacancies other than neutral charge state can only exist at negligible densities expected from the Boltzmann factor that has exponential dependence on formation energy^21^.

On the other hand, if monoclinic ZrO_2_ sample is in contact with a metal having a relatively large work function such as Ru which is generally the case for ZrO_2_ based ReRAMs^1,4,10^, the electron’s Fermi level is formed closer to the valence band maximum, and especially for interfacing with Ru electrode, most of O vacancies are thought to be formed at 3-fold O sites and would prefer to be +2q charged as can figure out in the figure. As a matter of fact, in the case of devices such as ReRAMs operating by externally applied electric field, charged O vacancies response to it inevitably play a crucial role for the performance of the device. Relative O vacancy formation energy determines the relative population between charge states O vacancies and is closely related with the efficiency of ReRAM, such as the on/off ratio. In this respect, it is clear that Ru electrode is one of the best choices for ZrO_2_-based ReRAM although there are many additional experimental considerations in choosing electrode materials in practice.

## Activation energy of O vacancy for various pathways

In Table 1, we summarize all the calculated activation barriers (E_A_) of neutral and positively charged O vacancies which were obtained in the frame of nudged elastic (NEB^25–27^) method for the paths shown in Figs. 2 and 3. NEB method assumes that migrating atom passes over a hypothetical elastic band that connects the two pre-obtained initial and final lattice configurations bearing specific types of O vacancies. The entire lattice, including this deliberately placed atom, is called an image, and each image (we used totally 8 images in the present calculations) is updated to the minimum energy configuration through ionic relaxation with GGA functionals and thus the entire band is updated to minimum energy variation and find the migration barrier (the more details about calculation method are explained in the Method section).

**Table 1:** Calculated activation energy (E_A_) of O vacancies for all the paths in monoclinic ZrO_2_.

Path		ⓐ	ⓑ	ⓒ	ⓓ	ⓔ	ⓕ	ⓖ	ⓗ	ⓘ	ⓙ
E_A_ (eV)	Neutral	2.35	1.85	1.37	2.64	2.54	2.74	2.43	2.07	2.25	2.49
	+1	1.48	1.54	1.00	1.71	1.26	1.85	2.16	1.26	2.01	2.40
	+2	0.87	1.20	0.70	1.06	0.92	1.13	1.99	0.88	2.04	2.73

For the paths having directional preference by the difference in formation energy between initial and final configurations (ⓖ~ⓙ), the larger value of the two are chosen. In this table, we can clearly notice there are overall tendency that as positive charge number increases, E_A_ decreases that can be generally understood by decreasing in overlap between outermost electronic orbitals^8,28^.

**Figure 5:**
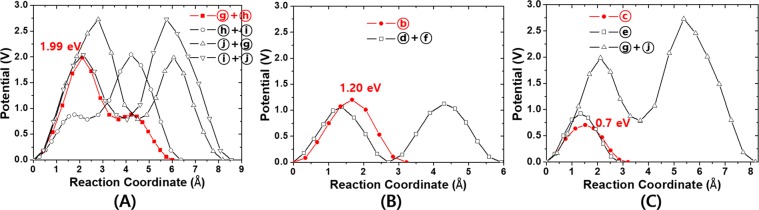
The energy variation during the neutral O vacancy migrations into three different crystallographic axes through the self-connected pathways shown in Fig. 3 in (**A**) (100), (**B**) (010), and (**C**) (001) directions. The paths with lowest activation barrier among the same category are plotted in red.

Keeping in mind that most of O vacancies in monoclinic ZrO_2_ interfacing with a large work function such as Ru, stays as +2q charged, in Fig. 5 we gather the total energy variations during +2q O vacancy migrations for the self-connected paths given in Fig. 3 categorized by the migration directions. In each direction, the path with the lowest E_A_ within the category is highlighted in red. We plot the lines to avoid near overlap between the lines by changing the direction of reaction coordinate. Because the activation energy is the same regardless of the reaction coordinate direction, so it does not matter the order of combination of paths as long as the common O site is correctly matched. It is clear if multiple paths are serially combined to form a self-connected path, the larger barrier determines E_A_ of the entire path^8,28^. Therefore, it can be interpreted that the migration of +2q O vacancies to the (100) direction (Fig. 5(A)) occurs primarily, while all the connected paths are combinations of two or more single paths, ⓖ+ⓗ (E_A_ = 1.99 eV) together with the higher barrier paths, ⓗ+ⓘ, ⓖ+ⓙ, and ⓘ+ⓙ of which E_A_ is represented by 2.04, 2.73, and 2.73 eV respectively, also contribute to the total migration barrier in the direction. And for (010) direction (Fig. 5(B)), dominant O vacancy migrations take place through ⓑ (E_A_ = 1.20 eV) while ⓓ+ⓕ (E_A_ = 1.13 eV) contributes negligibly, even with the smaller E_A_, due to the much larger formation energy of +2q 4-fold O vacancy than +2q 3-fold. Finally, for (001) direction (Fig. 5(C)), ⓒ path shows the lowest barrier (0.7 eV) and is the lowest in all directions. The importance of the ⓒ path that involves only two 3-fold O sites as an initial and final configurations is even much greater because the population of +2q 3-fold O vacancy is overwhelmingly larger than 4-fold as noticed from the formation energies shown in Fig. 4.

It is obvious if the interested surface of monoclinic ZrO_2_ is specified in one of these three crystallographic directions, the E_A_ would simply be determined by each representative path described above. However, in any arbitrary surface direction, O vacancy migrations have to be described as combination the three specific cases. As a matter of fact, it is known that monoclinic ZrO_2_ has a strong preference for (111) surface direction in terms of surface energy^25^ and thus if the exposed surface is (111) direction, O vacancies inevitably suffer all three crystal directional migrations described above. In this situation, on the whole, O vacancies are most likely to feel the largest barrier when migrating into (100) which has the largest E_A_ among three directions. This is thought to be the fundamental reason why the activation barrier reported in ref. ^19^ is about 2 eV.

However, because oxide volume of oxide-based ReRAMs that actual resistance switchings occur has a few nanometer thickness and tens of nanometers of diameter^8,26^, the situation stated above can be quite different. The near stoichiometric top layer deposited on the amorphous base layer is also likely to be amorphous as proved in ref. ^27^. In this situation, the top ZrO_2_ layer has various crystallographic orientations over the entire film surface. And then, an actual resistance changing region called ‘filament’ is formed within the top layer by causing electrical hard breakdown. This action triggers collective migration of positively charged O vacancies in the spot where O vacancies can most easily migrate, i.e. with lowest activation barrier. It is also likely that concentration of oxygen vacancies near top electrode interface is larger than bulk somehow due to non-negligible oxidation reaction and/or damages during deposition of top electrode. These increased O vacancies can possibly roles as seeds and promoters for the electrical breakdown in the top layer.

In fact, O vacancies may also migrate through grain boundaries in ZrO_2_ layer. But, considering that resistance switching of oxide-based ReRAMs is voluminal phenomenon^8,26^, even if O vacancy migration through grain boundary occurs during resistance switching, collective migration of O vacancies within a grain must be accompanied. In this respect, it is most likely that during forming process in ZrO_2_-based ReRAM, a filament is formed at the location where the crystal direction with the lowest activation barrier, (001) direction, happens to be aligned with the electric field so that +2q charged O vacancies migrate most easily.

The definitive total E_A_ must be expressed in statistical relation to the E_A_ of all the possible paths, taking into account the probability to be selected of each O migration path together with the diffusion constant of each path, and then, the effective total diffusion constant can be expressed generally by the following equation^19^.

2 $$\begin{array}{rcl}{D}_{total} & = & {D}_{tot}^{\ast }exp(-\frac{{E}_{A}^{tot}}{{k}_{B}T})\\  & = & \sum _{{\rm{k}}q}[{{\mathbb{P}}}_{{\rm{k}},q}({\mu }_{e},T)\times {D}_{{V}_{O}}^{\ast k,q}\exp (-{E}_{A}^{{\rm{k}},q}/{k}_{B}T)]\end{array}$$

where ${D}_{tot}^{\ast }$ and ${E}_{A}^{tot}$ are the total prefactor and activation energy respectively, ${{\mathbb{P}}}_{{{\rm {\bigcirc}\!\!\!{k}}},q}({\mu }_{e},T)$ is normalized probability of selection for path ⓚ which is involving migration of q charged O vacancy and ${D}_{{V}_{O}}^{\ast {\rm {\bigcirc}\!\!\!{k}},q}$ and ${E}_{A}^{{{\rm {\bigcirc}\!\!\!{k}}},q}$ are the corresponding diffusion prefactor and the activation barrier respectively. We are aiming to obtain ${E}_{A}^{tot}$ for O vacancy migration into a particular crystallographic direction and because while ${E}_{A}^{{{\rm {\bigcirc}\!\!\!{k}}},q}$s contribute as linear sum, whereas prefactors are logarithm sum, it is safe to ignore the difference in prefactors between individual paths.

Here, ${{\mathbb{P}}}_{{{\rm {\bigcirc}\!\!\!{k}}},q}({\mu }_{e},T)$ is again can be represented as the normalized product of the probabilities of existence of two O vacancies V_O1_ and V _O2_ that constitute path ⓚ as the initial and the final state of the migration derived from the probability theory of an event being selected that is the number of cases of selection divided by the total number of possible cases.

3 $${{\mathbb{P}}}_{{{\rm {\bigcirc}\!\!\!{k}}},q}({\mu }_{e},T)={M}_{{{\rm {\bigcirc}\!\!\!{k}}}}{p}_{{V}_{O1},q}({\mu }_{e},{\rm{T}}){p}_{{V}_{O2},q}({\mu }_{e},{\rm{T}}){(\sum _{{V}_{O},q,{\rm {\bigcirc}\!\!\!\!{m}}}{M}_{{\rm {\bigcirc}\!\!\!\!{m}}}{p}_{{V}_{O1},q}({\mu }_{e},{\rm{T}}){p}_{{V}_{O2},q}({\mu }_{e},{\rm{T}}))}^{-1},$$

where ${p}_{{V}_{O1},q}({\mu }_{e},{\rm{T}})$ and ${p}_{{V}_{O2},q}({\mu }_{e},\,{\rm{T}})$ are the probabilities of existence for V_O1_ and V_O2_ with charge q which are functions of formation energy and proportional to the respective Boltzmann factors^21^. *M*_ⓚ_ is the value indicating how many identical ⓚ paths exist around the same O site that equals 2 for ⓒ, ⓔ, and ⓖ and 1 for all the other paths. As can be clearly seen in Eq. (2), due to the exponential dependence of ${E}_{A}^{{{\rm {\bigcirc}\!\!\!{k}}},q}$ there is huge differences in the contributions to the final diffusion constant, even with very small differences in activation energy for each case.

There are two main reasons for focusing on activation energy instead of diffusion constant itself. One is that the predicted value from ReRAM operation currently we are interested in is activation energy, not diffusion constant. The value that only can be extracted from the ReRAM characteristic (operation model) is activation energy. The other reason is, the only exact value can be drawn by the NEB method is activation energy. In order to obtain prefactor information we should take into account the interaction with the phonons that exist in the lattice in thermal equilibrium, but theoretically, there is no other way than to guess by fitting with experiments which was the way ref. ^19^ employed.

## Crystallographic direction dependent activation energies of O vacancy

**Figure 6:**
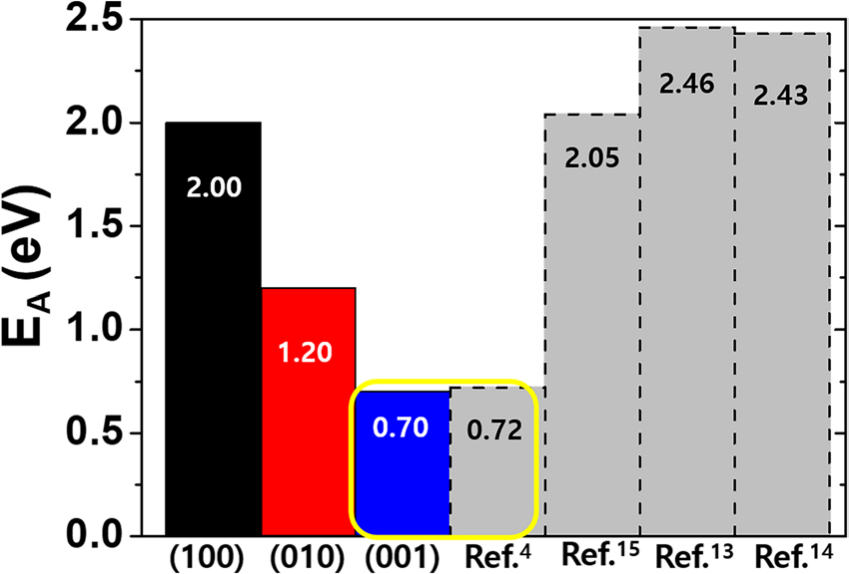
Comparisons between the present calculated O vacancy activation energies for the three crystallographic directions (solid black, red, and blue boxes for (100), (010), and (001) respectively) and the previously reported experimental measurements (refs. ^17,18^), the value derived from the ZrO_2_-based ReRAM switching model (refs. ^4,5^), and the previously reported theoretical value (ref. ^19^). The yellow box emphasizes the good agreement between the activation barriers of O vacancy from the switching model and the value of (001) direction.

As discussed in Fig. 3, if the Fermi level in monoclinic ZrO_2_ is at near the valence band maximum as expected for ZrO_2_-based ReRAM, most of O vacancies are formed at 3-fold sites and +2q charged, then Eqs. (2) and (3) become much simpler leaving only the summation for the paths and multiplicity factors. In Fig. 6, we present the obtained activation energies at temperature of 1200 K from Eqs. (2) and (3) in three different crystallographic directions for +2q O vacancies comparing with the activation barrier expected from the operational characteristic of ZrO_2_ ReRAM along with the previously reported experimental measurements for diffusion barrier. 1200 K of temperature is selected from the high temperature range of the previous experiments (ref. ^17,18^.) and the difference between 1200 K and 800 K from present calculation is less than 0.01 eV regardless of crystallographic direction.

In this figure, we can see that the calculated E_A_ in (001) direction (0.70 eV) is nearly coincides with the expected activation barrier of the ReRAM switching model (~0.72 eV, refs. ^4,5^) that clearly justifies our reasoning explained above. The resulting activation barrier in (001) direction is almost solely from ⓒ path with minor contribution of migrations through ⓔ and ⓖ+ⓙ owing to the much smaller formation energy of +2q charged 3-fold O vacancy. The previously reported theoretical result (~2.05 eV, ref. ^19^) is most likely originated from the +2q O vacancy migration into (100) direction with barrier of 2.00 eV. Since, in general, migration in any surface direction involves all three-axis migrations, the direction with largest barrier, (100) for monoclinic ZrO_2_, determines the total barrier. This is not always the case, as explained earlier, for a situation of a filament in oxide-based ReRAM we have been dealing with in the present study.

Meanwhile, the experimentally measured values (refs. ^17,18^), are by far larger than the present calculation. The experiment of ref. ^17^ was conducted with the monoclinic ZrO_2_ fabricated by passing hydrogen-water vapor mixture over the non-stoichiometric zirconium powder samples and the O activation energy was obtained by measuring the oxidation quantity of the sample. Ref. ^18^ measured conductivity of O isotope (^18^O) in ZrO_2_ sphere. Because the samples of both experiments are prepared with no particular surface direction, the experimental results must have had nature of bulk monoclinic ZrO_2_, in which the characteristics of all three crystallographic directions are mixed. The rather big discrepancy between the experimental values and the current calculation also might tell that they are not originated from +2q charged O vacancies, instead, it could be possible that neutral O vacancies with the much larger activation barriers might play major roles in the measurements which can be another subject of inquiry in the future.

## Discussion

It would be meaningful to add discussion of the applicability of this study to other non-bilayered ZrO_2_ ReRAMs^11–13^. In those reports, the I-V curves have opposite polarity to that of refs. ^2,4^ present study has referred to. First of all, it is necessary to distinguish whether the oxide layer of the devices can really be thought as a materially single layer or (unintentionally) can be regarded to be made up of multi-layers for somehow. However, considering that a bipolar ReRAM must have mobile ion storage layer that causes a change in resistance, except for CBRAM^27^ or the likes of using Schottky barrier formation at interface^29,30^, it is most likely that in the ReRAMs of refs. ^11–13^, the actual resistance switchings have taken place only in the part of the ZrO_2_ layer. Then there can arguably be mainly two cases: resistance change occurs near the bottom electrode or top electrode (present study). If for somehow more resistive layer is created at the bottom side, the opposite polarity of switching operation is explained so simply only by exchange of top and bottom electrodes. On the other hand, if resistance changing layer is located near top side, the story would be little bit complicated and then the question becomes if negatively charged metal ions (or O vacancy), or positively charged O ions (or metal vacancy) can exist in monoclinic ZrO_2_, however, according to the results of ref. ^24^, which have calculated the formation energies of various atomic defects in monoclinic ZrO_2_, it is very much unlikely. It is then can be concluded that ZrO_2_-based ReRAMs not designed with a bilayer structure are actually likely to have a bilayer structure in which only the location of the resistive change layer is reversed.

Thus, there is ample room for present study to be applied to non-bilayer ZrO_2_ ReRAMs. However, that would be only possible if the exact structural and material properties of each layer are exactly defined which is very difficult for ZrO_2_ ReRAMs that are not designed and fabricated as such in the first place.

In summary, we have presented the theoretical calculations of migration energy barrier of O vacancy in monoclinic ZrO_2_ based on the first principles method. Among the various charge states of O vacancies, we focused on +2q charge state, which are considered to be the largest population among all kinds of O vacancies in ZrO_2_-based ReRAM adopting an metal electrode with a large work function enough to make Fermi level locate at near valence band maximum. As is usually the case with oxide-based ReRAMs, in the case that the resistance change region (filament) is predetermined by electrical breakdown, the filament is formed at a spot where O vacancies can migrate most easily, namely, with the lowest migration barrier. We analyzed the migration barriers of +2q O vacancy by crystallographic directions of monoclinic ZrO_2_ and found that migration into (001) direction has the lowest barrier (~0.7 eV) which is in excellent agreement with the value from the ZrO_2_ ReRAM switching characteristic (~0.72 eV). In addition to that, we have figured out that O vacancy migration barrier in an arbitrary direction is represented by migration in (100) direction of which barrier (~2.00 eV) also almost coincides with the previously reported theoretical value (2.05 eV).

## Methods

The calculations are based on Kohn-Sham theory and the projector-augmented wave potentials as implemented in the Quantum ESPRESSO package^27^. For Zr the 5 s, 4d, and 5p orbitals were treated as valence states and the Perdew, Burke, and Ernzerhof (PBE) potential with a core radius of 3.07 a.u was used and for O, the PBE potential with a core radius of 1.55 a.u. was applied. Valence electron wavefunctions were expanded in a planewave basis set with a cutoff energy of 400 eV. We use, for all the calculations, the 2 ×2 ×2 supercell expanded from the unit cell of the monoclinic ZrO_2_ which comprises with 95 atoms with a single O vacancy. The ion relaxation calculations with an O vacancy that used for the initial and final structure of migration barrier calculations, were performed with the k-points generated with a mesh spacing 0.25 Å^−1^ applying generalized gradient approximated (GGA) exchange-correlation functional under Hellmann-Feynman force criterion of 0.02 eV/Å.

The migration pathway of the O vacancy and the corelated energy barrier were determined by finding the minimum energy path (MEP) from one lattice site to an adjacent site using a variation of the nudged elastic band (NEB)^29–31^ method as implemented in Quantum ESPRESSO. First, the two endpoint (initial and final) configurations of interested vacancy states were determined by separately optimized ion relaxation calculations. At these two states, it was assumed that the energies are in local minima and the forces applied at atoms are nearly zero. Since the MEP lies between two end states, firstly, a set of intermediate atomic configurations so called images are generated with constant spacing between the two endpoints, and this whole forms an ‘elastic band’. Then, finding the MEP is carried out, while keeping the two endpoints fixed, by nudging the elastic band little by little toward zero forces. In other words, for the images to reach the MEP, each image must be moved toward MEP by a vector of forces at each step. This forces mainly consist of interatomic forces due to displacement of atoms and an artificial spring force that added by connecting two adjacent images with a spring with a constant spring constant. This virtual spring force works only in a direction parallel to the line that connects the images and makes the images pass through the energy minimum that passes through the saddle point, not the real energy minimum and interatomic forces only act in the direction perpendicular to the band. More specifically, we used NEB climb method^29^ designed for one of images to be positioned at the saddle point of potential. This method minimizes the possibility of the NEB process reaching an incorrect path other than MEP. We adopted 8 images for all the calculations by means of a parallel calculation implementation of the Quantum ESPRESSO code, with each image assigned to a separate processor core.

## Data Availability

The data that support the findings of this study are available from the corresponding author upon reasonable request.
